# Simultaneous analysis of microbial identity and function using NanoSIMS

**DOI:** 10.1111/j.1462-2920.2007.01478.x

**Published:** 2008-03

**Authors:** Tianlun Li, Ting-Di Wu, Laurent Mazéas, Laurent Toffin, Jean-Luc Guerquin-Kern, Gérard Leblon, Théodore Bouchez

**Affiliations:** 1 Cemagref, Unité de recherche Hydrosystèmes et bioprocédésParc de Tourvoie, BP 44, 92163 Antony cedex, France; 2 Université Paris-Sud, Institut de Génétique et MicrobiologieOrsay, 91405 France; 3 Institut Curie, Laboratoire de Microscopie IoniqueOrsay, 91405 France; 4 INSERMU759, Orsay, 91405 France; 5 Laboratoire de Microbiologie des Environnements Extrêmes (UMR6197) IFREMER Centre de Brest, Technopôle Brest-IroiseBP70, 29280 Plouzané, France

## Abstract

Identifying the function of uncultured microbes in their environments today remains one of the main challenges for microbial ecologists. In this article, we describe a new method allowing simultaneous analysis of microbial identity and function. This method is based on the visualization of oligonucleotide probe-conferred hybridization signal in single microbial cells and isotopic measurement using high-resolution ion microprobe (NanoSIMS). In order to characterize the potential of the method, an oligonucleotide containing iodized cytidine was hybridized on fixed cells of *Escherichia coli* cultured on media containing different levels of ^13^C or ^15^N. Iodine signals could clearly be localized on targeted cells and the isotopic enrichment could be monitored at the single-cell level. The applicability of this new technique to the study of *in situ* ecophysiology of uncultured microorganisms within complex microbial communities is illustrated.

## Introduction

Since Antonie van Leeuwenhoek pioneered microscopic observation of microorganisms in the 17th century, direct examination of individual cells has remained one of the cornerstones of microbiology. More recently, in order to observe uncultured microbes, *in situ* hybridization of phylogenetically targeted probes has been developed (Giovannoni *et al*., 1988), revealing a surprising level of unseen prokaryotic diversity. Today, the challenge moves to the development of methodologies that allows the coupled examination of identity and function of uncultured microbes within complex ecosystems.

Lee and colleagues (1999) developed a new approach combining fluorescent *in situ* hybridization (FISH) and microautoradiography (MAR) for simultaneous detection of *in situ* identities, activities and specific substrate uptake profiles of individual bacterial cells within complex microbial communities. This approach can be advantageously coupled to stable isotope probing (SIP), another culture-independent method targeting extracted DNA or RNA and allowing the pre-identification of microbes catalyzing a given metabolic process in a complex environment (Radajewski *et al*., 2000; Manefield *et al*., 2002). By using these two techniques, Ginige and colleagues (2004) demonstrated a full-cycle rRNA and function analysis to establish a link between microbial phylogeny and physiology. However, MAR suffers from several limitations, including that some chemical elements do not have a radioactive isotope with suitable half-life time for use as a labelled tracer for FISH-MAR experiments. For example, important biological elements such as nitrogen or oxygen cannot be monitored using FISH-MAR.

In order to overcome these limitations, MAR could be advantageously replaced by secondary ion mass spectrometry (SIMS). The feasibility of using SIMS to measure isotopic composition of microbes at natural abundances (Orphan *et al*., 2001) or after isotopic enrichment (DeRito *et al*., 2005) had indeed been shown. Orphan and colleagues (2001) were also the first to have coupled SIMS with FISH to study anaerobic methane oxidizing archaea. However, in that case, microbial identity and isotopic composition were determined separately on two different instruments, which made exact mapping of samples necessary for SIMS analysis. Due to low lateral resolution, the isotopic measurement had to be performed specifically on pre-identified cell aggregates.

Latest major development in SIMS instrumentation (NanoSIMS 50^TM^) now allows applications in biology with a lateral resolution better than 100 nm (Slodzian *et al*., 1992; Guerquin-Kern *et al*., 2005). The capability of such instrument to measure isotopic composition makes it suitable to study environmental microbiology at the single-cell level (Lechene *et al*., 2006).

During the past several years, we have been developing a novel approach (SIMSISH, standing for SIMS *in situ* hybridization) allowing simultaneous analysis of microbial identity and function by NanoSIMS. The concept relies on performing FISH experiments, in which the fluorescent dye has been replaced by a molecule containing stable isotopes or elements rarely present in biomass (like halogens), in order to allow the detection of probe-conferred hybridization signals with NanoSIMS instrument. Using a single instrument, it might thus be possible to simultaneously detect the hybridization of the oligonucleotide probe revealing the phylogenetic identity of the targeted microbe and monitor *in situ*, at the single-cell level, its isotopic enrichment in various elements of biological interest (^13^C, ^15^N, ^18^O, . . .). The concept of this innovative methodology was recently mentioned in a perspective paper highlighting important developments in the field of microbial ecology (Kuypers and Jorgensen, 2007)*.*

## Results

**Fig. 1:**
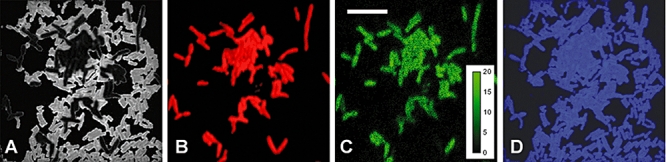
SIMSISH analysis of *E. coli* cells cultured on a medium containing 99% of ^13^C mixed with *B. subtilis* at natural isotopic abundance. *E. coli* were pre-hybridized with probe I_6_-Eub338-Cy3. Acquisition time was 25 ms pixel^−1^. A. ^12^C^−^ secondary ion image. B. ^13^C^−^ secondary ion image. C. ^127^I**^−^** secondary ion image, the green intensity scale represents the ^127^I^−^ counts number in NanoSIMS analysis. D. ^32^S^−^ secondary ion image, acquired to locate protein-containing biomass. Scale bar equals 5 μm.

The first step of the methodological development was to show the possibility of detecting iodized probe hybridization. For this purpose, ^13^C-labelled fixed cells of *Escherichia coli* (*E. coli*) were hybridized with bacterial probe I_6_-Eub338-Cy3 (5′-Cy3-GcTGccTcccGTAGGAGT-3′ c = 5-iodo-2′deoxycytidine, purposely synthesized by Proligo) and mixed with *Bacillus subtilis* (*B. subtilis*) grown on a natural isotopic composition medium. Figure 1 shows NanoSIMS images obtained from this mixture. Figure 1A shows the ^12^C^−^ secondary ions image, corresponding to the *B. subtilis* cells. Signals obtained on Fig. 1B (^13^C image) and Fig. 1C (^127^I image) are colocalized, which demonstrate that hybridization of the iodized probe was recorded on ^13^C-enriched *E. coli* cells only. A good contrast was obtained on ^127^I^−^ image, which demonstrated that hybridization of the probe could be detected with a satisfactory signal-to-noise ratio, showing that possible residual iodine signal levels from sample and from contamination by non-hybridized probe were below the detection limit of the instrument. The ^32^S image (Fig. 1D) was used in our study to have a general view of the total biomass.

In a second step, the possibility of single-cell quantitative isotopic composition measurement by NanoSIMS was evaluated. *E. coli* cells were prepared from culture media with different isotopic compositions in ^13^C or ^15^N (1.1%−10%−40%−70%−99% of ^13^C, 0.4%−10%−40%−70%−99% of ^15^N). Only *E. coli* cells cultured on the medium containing 40% of ^13^C or ^15^N were hybridized with I_6_-Eub338-Cy3 probe. After NanoSIMS observation, an image analysis procedure (see *Experimental procedures*) was applied in order to determine the ^13^C or ^15^N enrichment level of the observed cells. The isotopic compositions determined directly from the NanoSIMS observation, at single-cell level, were in good overall agreement with the isotopic compositions measured by elemental analysis-isotope ratio mass spectrometry (EA-IRMS) for each cell culture (Table 1).

**Table 1:** Comparison of isotopic composition measurement by NanoSIMS and by IRMS (mean value with standard error).

Isotopic composition of the culture media		Isotopic abundance in raw *E. coli* cell pellets analysed by IRMS (duplicated analysis)		NanoSIMS analysis on a set of single cells (10 replicate analysis)	
		
^13^C	^15^N	^13^C atom %	^15^N atom %	^13^C abundance ^13^C/(^12^C+^13^C)	^15^N abundance ^15^N/(^14^N+^15^N)
Natural	Natural	1.10 ± 0.00%	0.39 ± 0.00%	1.1 ± 0.03%^a^	0.40 ± 0.01%
10%	Natural	8.85 ± 0.09%	0.37% ± 0.00%	9.6 ± 0.17%^a^	–
40%	Natural	33.99 ± 0.08%	0.37% ± 0.00%	32.6 ± 0.22%^b^	–
70%	Natural	58.12 ± 0.47%	0.38% ± 0.00%	58.0 ± 0.41%^a^	–
99%	Natural	–	–	86.8 ± 0.63%^a^	–
Natural	10%	1.10 ± 0.00%	9.25 ± 0.10%	–	10.1 ± 0.17%
Natural	40%	1.10 ± 0.00%	36.46 ± 0.21%	–	38.9 ± 0.19%^b^
Natural	70%	1.10 ± 0.00%	63.39%	–	66.5 ± 0.13%
Natural	99%	–	–	–	94.2 ± 0.16%
50%	50%	49.10 ± 0.01%	44.21 ± 0.25%	42.1 ± 0.35%^b^	39.8 ± 0.16%^b^

a Analysis performed on fixed cells.

b Analysis performed on fixed and pre-hybridized cells. Other values were obtained on unfixed and non-hybridized cells.

^13^C and ^15^N isotopic composition of the different pure *E. coli* cell cultures were determined by IRMS. NanoSIMS analysis data were obtained by image analysis procedure.

**Fig. 2:**
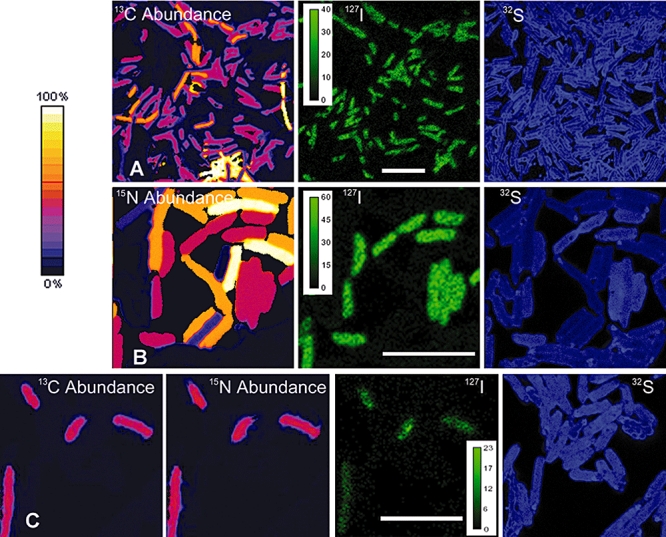
SIMSISH analysis for bacterial culture samples. A. Mixture of different *E. coli* cell cultures grown, respectively, on media at 1.1%, 10%, 40%, 70% and 99% in ^13^C. *E. coli* cell culture grown on medium at 40% in ^13^C was pre-hybridized by probe I_6_-Eub338-Cy3. B. Mixture of *E. coli* cell cultures grown on media from different isotopic composition (respectively 0.4%, 10%, 40%, 70% and 99% in ^15^N). The *E. coli* cell culture grown on the medium containing 40% of ^15^N was pre-hybridized by probe I_6_-Eub338-Cy3. C. *E. coli* cell culture grown on the medium containing 50% of ^13^C and 50% of ^15^N was pre-hybridized by probe I_6_-Eub338-Cy3. For the three series, image in green represents ^127^I^–^ secondary ion image (the green intensity scale represents the ^127^I^−^ counts number in NanoSIMS analysis), and image in blue represents ^32^S^−^ secondary ion image, acquired to locate protein-containing biomass. The colour scale on the left allows us to easily recognize cell isotopic abundance (each colour corresponds to a 5% increment). Scale bar equals 5 μm.

Figure 2 shows results of hybridization and isotopic measurement obtained on three different mixtures of *E. coli*. Original greyscale images obtained for different elements were displayed with different colours. ^32^S secondary ion image corresponding to the whole biomass was displayed using a blue-coloured intensity scale, while a green one was used to show the iodine distribution associated to hybridized cells. On the left column images, stable isotope abundances were displayed in a linear scale using a colour lookup table (fire-type) to allow easy visualization of the different levels of isotope abundances. The ^127^I^−^ signal was recorded only on targeted cells (purple cells at 32.6% in ^13^C on Fig. 2A and red cells at 38.9% in ^15^N on Fig. 2B) as expected from the design of the experiment.

The possibility to observe several elemental isotopic compositions at the same time and at the single-cell level was then questioned. Thus, double-labelling experiment of *E. coli* grown on culture medium containing 50% ^13^C and 50% ^15^N were performed. Cells were fixed and hybridized with probe I_6_-Eub338-Cy3 and mixed with non-hybridized *E. coli* cells at natural isotopic composition as control. Simultaneous measurement of ^15^N and ^13^C abundance was determined on enriched cells (Fig. 2C). As expected, the control cells showed natural ^13^C and ^15^N isotopic compositions and no signal for ^127^I. The results of ^13^C and ^15^N abundance given by the image analysis procedure for hybridized cells are presented in Table 1.

**Fig. 3:**
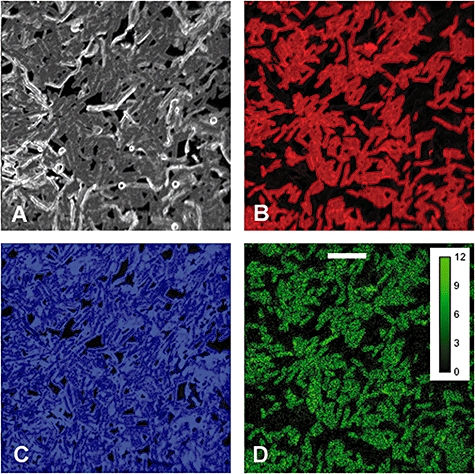
SIMSISH analysis of *E. coli* grown on a medium containing 99% of ^13^C mixed with an environmental sample. *Escherichia coli* cells were pre-hybridized with probe I_6_-Eub338-Cy3. All images are 30 μm × 30 μm. Acquisition time was 25 ms pixel^−1^. (A) ^12^C^−^ secondary ion image, (B) ^13^C^–^ secondary ion image, (C) ^32^S^−^ secondary ion image, (D) ^127^I^−^ secondary ion image (the green intensity scale represents the ^127^I^−^ counts number in NanoSIMS analysis). Scale bar equals 5 μm.

The potential of SIMSISH method to analyse *in situ* the ecophysiology of uncultured microbes within complex microbial communities was illustrated using samples from a Municipal Solid Waste (MSW) batch bioreactor. Such environmental samples may contain high levels of impurities. To evaluate the level of iodine background, *E. coli* cell culture labelled in ^13^C was hybridized with probe I_6_-Eub338-Cy3. Hybridized cells were mixed with the fixed environmental sample and observed using NanoSIMS. Observations showed that the ^127^I^−^ signal was perfectly colocalized with ^13^C-labelled microorganisms (Fig. 3) while no significant iodine background was detected elsewhere. These results illustrated the fact that hybridization of an iodized probe allowed a specific detection of probe-targeted cells in our environmental sample with a sufficient signal-to-noise ratio on the iodine image. The specificity of I_6_-Eub338-Cy3 probe was then checked on an artificial mixture of ^15^N-labelled *E. coli* and *Pyrococcus abyssi* at natural isotopic composition. For this experiment, I_6_-Eub338-Cy3 probe was applied directly to the mixture. NanoSIMS analysis showed that iodine signal was colocalized with ^15^N signal, indicating that only *E. coli* cells were hybridized by the probe (Fig. S1). The ^127^I signal in *P. abyssi* was detected at the same level as the background. This result demonstrated that there was no unspecific binding of the halogenated probe.

**Fig. 4:**
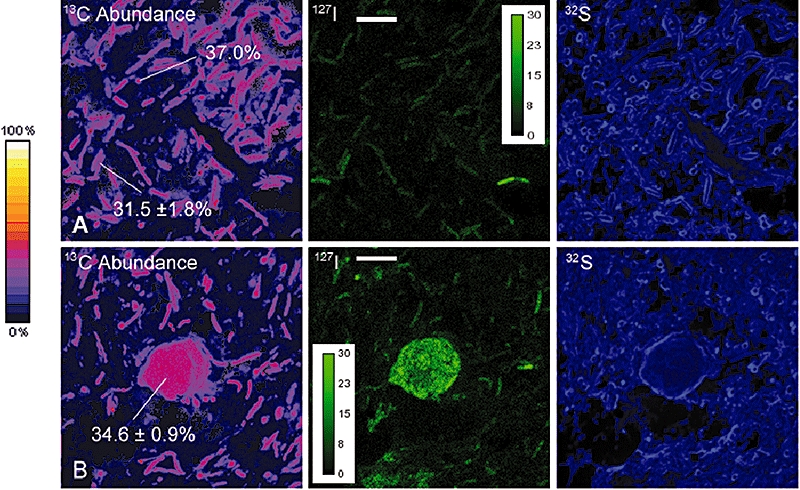
SIMSISH analysis for environmental samples. Sample taken from a MSW bioreactor where ^13^C-methanol was added. A. Sample was hybridized with bacterial probe I_6_-Eub338-Cy3. B. Sample was hybridized with archaeal probe I_10_-Arc915-Cy3. ^13^C abundances of bacterial and archaeal populations have been specifically measured at 31.5 ± 1.8% and 34.6 ± 0.9% respectively. The colour schemes are the same as described in Fig. 2. Scale bar equals 5 μm.

Then, we applied the bacterial probe (I_6_-Eub338-Cy3) or archaeal probe (I_10_-Arc915-Cy3, 5′-GTGcTcccccGccAATTccT-3′, c = 5-iodo-2′deoxycytidine, Proligo) to two fixed samples from the same bioreactor after addition of ^13^C-methanol (see *Experimental procedures* for details). NanoSIMS analysis of non-hybridized controls confirmed that ^127^I^−^ signal was under the detection threshold. In the sample hybridized with I_6_-Eub338-Cy3 probe, signal was detected only on rod-shaped cells (Fig. 4A). These cells exhibited a homogeneous ^13^C isotopic composition (at 31.5 ± 1.8%, *n* = 10). Some coccoid-like cells also showed a ^13^C isotopic composition at 37.0%, but displayed no detectable ^127^I^−^ signal, suggesting that microbes not targeted by EUB338 probe were also involved in methanol metabolism. In the sample hybridized with I_10_-Arc915-Cy3 probe, ^127^I^−^ signal was observed from a cluster of cells (7 μm diameter, Fig. 4B), and their ^13^C isotopic composition (at 34.6 ± 0.9%, *n* = 10) showed that these archaea had assimilated carbon originating from labelled methanol. These observations confirm that SIMSISH represents a novel and elegant approach to directly observe isotope assimilation by 16S rRNA-probe-identified microorganisms in complex environmental samples.

## Discussion

Two halogenated oligonucleotide probes and different sample deposition procedures were initially tested for optimization of the developed methodology. Evaluation of unspecific background contamination level was carried out for each halogen by using non-hybridized controls. In preliminary experiment, a ^19^F-labelled probe was used (labelled with Oregon green dye containing four atoms of fluorine). It resulted in excessively high unspecific background signal (data not shown). We finally selected iodine as a suitable atom for NanoSIMS imaging studies not only because it gave good signals (due to strong ionization yield), but also because it was not detected in any of non-hybridized samples that we analysed.

The choice of appropriate sample supporting material is also critical to achieve good signal-to-noise ratio for biological sample observation. We tested several common support material used in NanoSIMS studies as polished stainless steel plate, carbon-coated TEM (transmission electron microscopy) grid and silicon wafers. In our hand, silicon wafer performed better than other material tested. Both stainless steel and TEM grid contain detectable trace of iodine while such background was hardly detectable on cleaned silicon chips during experiments. Moreover, the carbon coating on TEM grid may interfere with the sample during the carbon isotopic composition measurement.

SIMSISH represents a novel methodology for simultaneous analysis of microbial identity and function. Compared with Quantitative-MAR (Q-MAR) (Nielsen *et al*., 2003), which presents a quantitative approach of visualizing and measuring radioactive isotopes, NanoSIMS instrument does not necessarily require the use of radioactive isotopes. It thus offers the possibility to use stable isotopes such as ^15^N or ^18^O for example. It features a higher imaging resolution (below 100 nm, roughly 10-fold higher than MAR) (Nielsen *et al*., 2003) and sensitivity (e.g. for ^14^C roughly 1000-fold higher than Q-MAR) (Williams, 2006). Furthermore, by measuring directly elemental isotopes in parallel (e.g. ^12^C^−^ and ^13^C^−^), the NanoSIMS instrument offers the unique possibility to determine isotopic ratios. The precision of this measurement has indeed already been demonstrated (Messenger *et al*., 2003). In contrast, Q-MAR needs an internal standard of bacteria with known radioactive isotopic composition, which makes the comparison of quantitative data from different studies problematic (Pernthaler and Amann, 2005). However, in the SIMSISH procedure, both sample fixation and probe hybridization could modify the cell isotopic composition by introducing exogenous carbon and nitrogen at natural abundance. These effects might explain some discrepancies observed in Table 1 between NanoSIMS and IRMS analysis, for which cell pellets were analysed directly without fixation and hybridization. Determining *in situ* actual cell isotopic composition with NanoSIMS would require further developments on fixation procedures (e.g. cryo-fixation) and on the evaluation of probe hybridization effect on the cell isotopic composition measurement.

Low signal intensity is another frequent problem for *in situ* hybridization, especially in environmental samples where ribosome copy numbers per cell are too low to provide sufficient hybridization signals. In our SIMSISH experiments, hybridized *E. coli* cells after overnight incubation exhibited sufficient iodine signal to be distinguished from background. Under the analytical conditions applied, iodine signal of hybridized *E. coli* ranged from 10 to 60 counts per pixel (Figs 1–3). On Fig. 2C, these counts resulted in signal-to-noise ratio ranging from 18 to 40. Lower values might result in insufficient signal-to-noise ratios. We therefore consider that a probe containing six atoms of iodine was necessary in our case to obtain reliable detection of probe hybridization.

We believe that the application of SIMSISH can be extended to a wide range of isotopes, which could serve either as biogeochemical tracers or as nucleic acid labels. With the use of proper isotopic tracers, this new methodology thus lends itself to address important issues in environmental microbiology related to the study of carbon and nitrogen flow in environmental ecosystems such as elemental and molecular exchanges between microbial community members, fixation and accumulation processes. Besides the isotope ^127^I, other elements (^80^Br, ^15^N, ^18^O, ^2^H, . . .) may be suitable candidates for nucleic acid probe labelling. Different isotope-labelled probes with overlapping specificities could then be used simultaneously to identify individual microbial cells following a Top-to-Bottom approach (Amann *et al*., 1995). We consider that SIMSISH technique has an enormous potential to document *in situ* the functions of uncultured microbes within complex environmental ecosystems. Combination of SIP-full-cycle rRNA analysis and SIMSISH offers an elegant solution to decipher networks of biogeochemical processes catalysed by uncultured microorganisms within complex microbial communities.

## Experimental procedures

### Bacterial strains and culture conditions

*Escherichia coli* JM109 (Promega) and *B. subtilis* 168m were grown in a defined medium containing per litre of distilled water 1 g of NH_4_Cl, 0.2 g of MgSO_4_·7H_2_O, 6 g of NaH_2_PO_4_·H_2_O, 3 g of K_2_HPO_4_, 0.5 g of NaCl and 0.1 g of CaCl_2_, supplemented with 10 mg ml^−1^ glucose as the sole organic carbon source. For ^13^C or/and ^15^N labelling, glucose or/and unlabelled NH_4_Cl were substituted by ^13^C_6_-glucose (99 atom % at ^13^C, Cambridge isotopic laboratory, UK) or/and ^15^NH_4_Cl (99 atom % at ^15^N, Cortecnet, France). All strains were cultivated aerobically at 37°C and harvested in stationary phase after 20 h of incubation. Series of *E. coli* were cultivated in the previously described medium containing incremental isotopic enrichment of ^13^C or ^15^N (10%, 40%, 70%, 99%) as sole carbon or nitrogen source respectively. ^13^C, ^15^N double-labelled *E. coli* cells were produced in the previously defined culture medium containing isotopic enrichment of 50% ^13^C and 50% ^15^N as sole carbon and nitrogen source.

*Pyrococcus abyssi* strain GE5 was grown anaerobically in 100 ml serum bottles containing 40 ml of medium referred to as ‘YPS’ (Erauso *et al*., 1993), sealed with blue butyl rubber stoppers (Bellco, Vineland, NJ, USA). Cultures were incubated in a rotary shaker (200 r.p.m.) at 90°C.

### EA-IRMS analysis of cell pellets

All cultured cells were pelleted at 11 000 *g* for 10 min at 4°C. The pellets were washed once with sterile ultrapure water and pelleted again at 11 000 *g* for 10 min. Pellets were dried overnight at 55°C. For each pellet, two samples of about 150 μg each were transferred to ultrapure tin container (Thermo). They were then subjected to isotopic analysis (^13^C and ^15^N) by EA-IRMS from Thermo Electron (Germany).

### Environmental samples

A labscale MSW bioreactor (1L) was incubated at 35°C under anaerobic conditions as described previously (Vigneron *et al*., 2005). At day 200, when the majority of biodegradable organic waste mass was decomposed, leachate residues were dispensed in an anaerobic chamber into 100 ml vials each of them containing 50 ml of mixture. In order to reveal the mechanism of methanol assimilation in this ecosystem, ^13^C methanol (99 atom % at ^13^C, Cambridge isotopic laboratory, UK) was injected in one vial (at a final concentration of 5 mg ml^−1^) and incubated under anaerobic conditions at 35°C, without shaking in the dark. Twenty-five days after the isotope tracer injection, 2 ml of liquid was sampled and fixed for SIMSISH analysis.

### Cell collection, fixation and *in situ* hybridization

Culture samples collected in stationary phase (1 ml) or environmental samples (2 ml) taken from bioreactor were pelleted at 11 000 *g* for 10 min at 4°C. The pellets were washed once with 1× phosphate-buffered saline (PBS, Sigma) and re-suspended in 200 μl of 1× PBS and 600 μl 4% paraformaldehyde (Sigma) as fixative. After 3 h of incubation at 4°C, tubes were centrifuged (11 000 *g*, 10 min) and pellets were washed once again with 1× PBS and re-suspended in 500 μl of 1× PBS and 500 μl of pure ethanol. Fixed cells were stored at −20°C. For *in situ* hybridization, 10–100 μl of fixed sample was mixed with equal volume of 0.02 M EDTA in order to induce partial deflocculation. Cells were pelleted by centrifugation (11 000 *g*, 10 min, 4°C) and washed in 400 μl of hybridization buffer (0.9 M sodium chloride, 20 mM Tris-HCl, 0.1% SDS and 20% of formamide). The resulting cell suspension was subjected to vigorous vortex (1 min). Cells were recovered by centrifugation and re-suspended in 20 μl of pre-heated hybridization buffer. Two microlitres of probe (50 ng μl^−1^) was added and the suspension was incubated during 2 h at 46°C. Cells were then recovered by centrifugation (11 000 *g*, 10 min) and washed for 15 min in wash buffer (0.215 M sodium chloride, 20 mM Tris-HCl, 5 mM EDTA, 0.1% SDS) at 48°C. Finally, the cells were centrifuged for 10 min at 11 000 *g* and re-suspended in 50 μl of sterile ice-cold ultrapure water.

### NanoSIMS acquisition and image analysis procedure

For NanoSIMS analysis, 1 μl of sample (1:50 dilution for pure culture) was spread on 7 mm × 7 mm high-purity silicon chips (Silicon Quest International) cleaned with ultrapure water and absolute ethanol. After drying in a vacuum oven at 55°C overnight, samples were then introduced into a NanoSIMS-50 instrument (CAMECA, Gennevilliers, France) equipped with caesium ion source with a local vacuum level less than 7 × 10^−8^ Pa surrounding the sample during analysis.

The elements surveyed were carbon, sulfur, iodine and nitrogen. The first three elements can be easily detected with good sensitivity using a Cs^+^ primary ion beam. Although nitrogen cannot be detected directly as N^−^, excellent sensitivity for this element can be achieved by detecting the CN^−^ cluster ion. For the present study, by using a Cs^+^ primary ion beam tightly focused to a typical probe size of about 100–150 nm in diameter, we targeted up to five of the secondary ion species (among ^12^C^−^, ^13^C^−^, ^12^C^14^N^−^, ^12^C^15^N^−^, ^13^C^14^N^−^, ^32^S^−^, ^127^I^−^), which were collected on separate detectors (electron multipliers, EM) on the parallel detection system. Each detector was placed at the exit of the mass spectrometer at a specific position according to the radius of the ions in the magnetic sector.

The area of interest was first selected by rapid survey with detection of iodine signal on detector EM #4 at high-mass resolving power condition and ^32^S^−^ signal on detector EM #3 in order to localize hybridized cells in the whole biomass as described in Table 2 which presents the different detectors settings used. During the same scan, two other ion species (EM #1 and #2) were also recorded to localize isotopically enriched cells.

**Table 2:** Detector set-up of the NanoSIMS parallel detection system.

		Detector configuration				
		
Purpose	Element of interest	EM #1	EM #2	EM #3	EM #4	EM #5
Survey and iodine detection	^13^C	^13^C^−^	^12^C^14^N^−^	^32^S^−^	^127^I^−^	
		^12^C^−^	^13^C^14^N^−^			
	^15^N	^12^C^−^	^12^C^15^N^−^	^32^S^−^	^127^I^−^	
	^13^C, ^15^N	^13^C^−^	^12^C^15^N^−^	^32^S^−^	^127^I^−^	
		^12^C^−^	^13^C^15^N^−^			
Isotopic abundance measurement	^13^C %	^12^C^−^	^13^C^−^	^12^C^14^N^−^	^13^C^14^N^−^	^32^S^−^
	^15^N %			^12^C^14^N^−^	^12^C^15^N^−^	^32^S^−^
	^13^C %, ^15^N %	^12^C^−^	^13^C^−^	^12^C^14^N^−^	^12^C^15^N^−^	^32^S^−^

Precise abundance determination required a second acquisition with different detectors settings (Table 2). In fact, the abundance of ^15^N can only be obtained by parallel detection of ^12^C^14^N^−^ and ^12^C^15^N^−^ while the one for ^13^C was usually obtained by parallel detection of ^12^C^−^ and ^13^C^−^. In either case, one needs the detection of two adjacent species as ^12^C^14^N^−^ and ^12^C^15^N^−^, or ^12^C^−^ and ^13^C^−^. Unfortunately, the limitation in mechanical spacing of two adjacent detectors of the current system does not allow these measurements when iodine was selected as the highest mass species. In all cases, ^32^S^−^ was monitored not only to provide a general view of the whole biomass but also to allow perfect correlation between the survey and abundance measurements. For precise and reproducible determination of isotopic abundance, the detectors EM #1 to #4 were carefully adjusted by checking the respective response to the ion species of interest so as to minimize fractionation during ion counting on separate detectors. It is worth noticing that the ^13^C abundance can also be measured by detecting ^12^C^14^N^−^ and ^13^C^14^N^−^. When they are measured in parallel with ^12^C^−^ and ^13^C^−^, one can calculate the abundance by two independent approaches so as to check the proper adjustment for all the four detectors involved.

The two neighbouring species ^12^C^15^N^−^ and ^13^C^14^N^−^ (with a relative mass difference of 4273, M/ΔM) were resolved at high-mass resolution condition (Guerquin-Kern *et al*., 2005). For the detection of ^13^C^−^, care has been taken so as to discriminate the isobaric species ^12^C^1^H^−^. High-mass resolving power was also applied to the detection of ^127^I^−^ due to the possible mass interference from an unidentified ion species observed in biological samples.

During analysis, the primary beam was scanned point by point with a definition of 256 × 256 pixels, over an area that ranges from 10 × 10 to 50 × 50 μm. Sets of images (each one corresponding to a selected secondary ion) were recorded. For rapid survey over large areas, the primary current was 5 pA with a spot size of about 150 nm and the typical counting time was 5–10 ms per pixel (dwell time). Further, for abundance determination over reduced area, the spot size was turned down to 100 nm with current of 1.5 pA while the typical counting time was extended to 15–30 ms per pixel. The ion counting time was kept constant between ions of the same elemental composition (e.g. ^12^C^14^N^−^ and ^12^C^15^N^−^) for isotope ratio measurements.

For *in situ* determination of isotopic composition, image processing was carried out using ImageJ, a Java-based free software (W.S. Rasband, ImageJ, US National Institutes of Health, Bethesda, MD, USA, http://rsb.info.nih.gov/ij/, 1997–2006). The abundance of ^13^C was determined with the following formula:

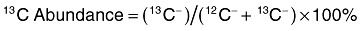

In practice, image calculator operator in ImageJ allows the above calculation done for each pixel of the whole image. Therefore, the distribution of ^13^C abundance can be obtained by dividing the ^13^C^−^ image by the sum of ^12^C^−^ image and ^13^C^−^ image. Similarly, the determination of the abundance of ^15^N was deduced by using the following formula:

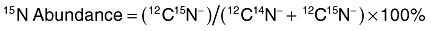

Again, image calculator operator was used to obtain the distribution of ^15^N isotopic composition by dividing the ^12^C^15^N^−^ image by the sum of ^12^C^14^N^−^ image and ^12^C^15^N^−^ image. The resulting abundance distribution is displayed in 32-bit floating point greyscale. The function ‘Measure’ in ImageJ provides a mean value for a selected area. Isotopic composition of single cells was taken as the mean value measured in central region of the cell. The isotopic compositions reported in Table 1 are mean values calculated from at least 10 different zones. Colour bar can also be used to increase artificially the differentiation for slight change in abundance. Finally, to avoid confusion in the resulting distribution of the pixels outside bacteria, for each set of images, a mask was generated based on the image of ^32^S^−^ as all the biomass exhibits high sulfur content.

## Supplementary material

The following supplementary material is available for this article online:

Fig. S1 SIMSISH analysis of artificial mixture of ^15^N-labelled *E. coli* and *Pyrococcus abyssi* at natural isotopic composition. The I_6_-Eub338-Cy3 probe was applied directly to the mixture. All images are 10 μm × 10 μm. Acquisition time was 25 ms pixel^−1^. (A) ^12^C^14^N^–^ secondary ion image, (B) ^32^S^–^ secondary ion image, (C) ^127^I^–^ secondary ion image, (D) superimposition of images (A), (B) and (C). Scale bar equals 2 μm.

This material is available as part of the online article from http://www.blackwell-synergy.com
