# Supplementary material for: Simultaneous analysis of microbial identity and function using NanoSIMS
Source: Environ Microbiol. 2008 Mar;10(3):580–8. doi: 10.1111/j.1462-2920.2007.01478.x (PMC2253709; doi:10.1111/j.1462-2920.2007.01478.x)
Supplement: Fig. S1 — SIMSISH analysis of artificial mixture of 15N-labelled E. coli and Pyrococcus abyssi at natural isotopic composition. The I6-Eub338-Cy3 probe was applied directly to the mixture. All images are 10 μm × 10 μm. Acquisition time was 25 ms pixel−1. (A) 12C14N– secondary ion image, (B) 32S– secondary ion image, (C) 127I– secondary ion image, (D) superimposition of images (A), (B) and (C). Scale bar equals 2 μm. [file emi0010-0580-sd1.doc]

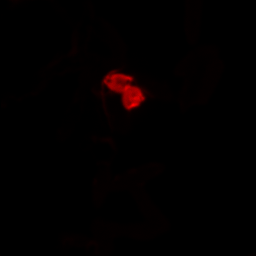

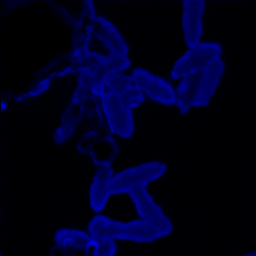


# A B

**C D**


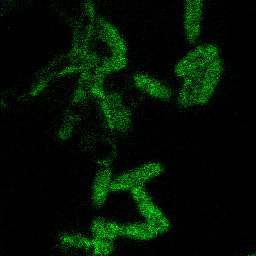

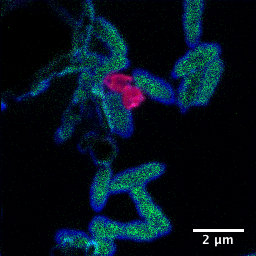


Figure S1: SIMSISH analysis of artificial mixture of ^15^N‑labeled *E. coli* and *Pyrococcus abyssi* at natural isotopic composition. The I_6_-Eub338-Cy3 probe was applied directly to the mixture. All images are 10 µm x 10 µm. Acquisition time was 25 ms/pixel. (A) ^12^C^14^N^-^ secondary ion image, (B) ^32^S^-^ secondary ion image, (C) ^127^I^-^ secondary ion image, (D) overlapping of Image A B and C. Scale bar equals 2 µm.
